# Age-Related Time-to-Onset Analysis of Drug-Associated Erythema Multiforme Using the Japanese Adverse Drug Event Report Database

**DOI:** 10.7759/cureus.114044

**Published:** 2026-08-06

**Authors:** Hideyuki Tanaka, Satoshi Nakao, Kana Sugishita, Tomofumi Yamazaki, Seiichiro Ito, Koichi Kageyama, Mugita Sato, Yoko Ino, Kazuhiro Iguchi, Mitsuhiro Nakamura

**Affiliations:** 1 Laboratory of Drug Informatics, Gifu Pharmaceutical University, Gifu, JPN; 2 Laboratory of Pharmaceutical Health Care and Promotion, Gifu Pharmaceutical University, Gifu, JPN; 3 Laboratory of Community Pharmacy, Gifu Pharmaceutical University, Gifu, JPN

**Keywords:** adverse drug event, erythema multiforme (em), jader, pharmacovigilance database, time-to-onset analysis

## Abstract

Introduction: Erythema multiforme (EM) is a rare immune-mediated skin condition often triggered by infections, drugs, or autoimmune diseases. Age-related differences in drug exposure, comorbidities, and biological factors may affect adverse event reporting patterns; however, age-related patterns in EM onset remain unclear. This study investigated age-related patterns in reported time-to-onset of EM.

Methods: The observation period was defined as 1-180 days from the initiation of drug administration. Kendall’s tau-b rank correlation analysis was used to evaluate the association between ordered age categories and reported time to onset. The Kaplan-Meier method with a log-rank test was used to compare EM onset profiles between two age groups (<60 and ≥60 years).

Results: A positive association was observed between age category and reported time-to-onset (Kendall’s tau-b = 0.188, p < 0.001). The median time-to-onset was 10.0 days in both men and women, although the Kaplan-Meier curves differed significantly by sex (log-rank p = 0.012). The median time-to-onset was 8.0 (3.0-13.0) days for those aged <60 years and 12.0 (7.0-24.5) days for those aged ≥60 years (log-rank p < 0.001).

Conclusions: Older age was associated with longer reported time-to-onset of EM, suggesting age-related differences in reported EM onset patterns in a spontaneous reporting database.

## Introduction

Erythema multiforme (EM) results from an immune-mediated reaction that primarily affects the skin and sometimes the mucosa [1-3]. Lesions usually erupt over a three- to five-day period and, in some cases, result in mild pruritus or a burning sensation [4]. EM lesions appear symmetrically on the extremities (especially on the extensor surfaces) and spread centripetally. The prognosis of mild EM is typically favorable, with spontaneous recovery in many cases.

EM is primarily caused by cell-mediated immune responses, with the main causes being infections, drugs, vaccines, and autoimmune diseases [1-4]. Most cases of EM are a consequence of infectious diseases, predominantly herpes simplex virus type 1, followed by *Mycoplasma pneumoniae*, the latter being particularly prevalent in children [5,6]. Many drugs have been associated with EM, with the most common ones being nonsteroidal anti-inflammatory drugs, antiepileptics, and antibiotics [1-3].

EM affects less than 1% of the population and is most common in young adults (younger than 40 years) [3,4]. Age-related changes in immune function, including immunosenescence, have been associated with increased susceptibility to infection and altered vaccine responses in older adults [7-9]. Sex differences in immune function have also been reported: women generally mount stronger immune responses to infection and vaccination than men but are more susceptible to certain autoimmune diseases [10,11]. Because EM is considered an immune-mediated reaction, these observations provide relevant context for evaluating age- and sex-related patterns in the reported time-to-onset of EM.

In clinical and pharmacovigilance settings, the time-to-onset after drug exposure is an important consideration when evaluating suspected drug-associated EM, particularly in patients receiving multiple drugs or those with possible nondrug triggers such as infections. Previous studies on the time-to-onset of cutaneous adverse drug reactions were often limited to specific drug classes, such as antiepileptics [12], or focused on specific phenotypes [13]. A recent study has suggested that latency may vary according to the clinical phenotype and implicated drug [14]. Since drug prescribing patterns differ significantly by age [15], age-related differences in time-to-onset may be influenced by differences in the types of drugs prescribed.

This study analyzed the Japanese Adverse Drug Event Report (JADER) database, which provides detailed data on the timing of adverse events (AEs), for a time-to-onset analysis. Using the JADER database, this study aimed to evaluate age- and sex-related patterns in the reported time-to-onset of EM. To account for this potential influence, we also examined EM onset patterns stratified by Anatomical Therapeutic Chemical (ATC) classification.

## Materials and methods

Data source

The JADER database from April 2004 to September 2024 was downloaded from the Pharmaceuticals and Medical Devices Agency (PMDA) website of the Japanese Regulatory Authority [16]. All data from the JADER database were cleaned and anonymized by the PMDA. The JADER database consists of four data tables: patient demographic information (DEMO), drug information (DRUG), adverse events (REAC), and primary disease information (HIST). In the DRUG table, each drug is classified as "suspected," "concomitant," or "interaction." For the present analysis, the DEMO, DRUG, and REAC tables were merged using the report identification number. EM records were extracted from the REAC table, and duplicate records with the same report identification number, preferred term (PT), and AE onset date were removed. Only drugs classified as suspected were retained. No missing values were imputed. Each analysis included records with available data for the variables required for that analysis. Records with unknown sex were retained in the overall analyses but excluded from the sex-stratified analyses. The ATC classification system was used to categorize drugs based on their anatomical and therapeutic properties [17].

Definition of adverse events

AEs in the JADER database were defined based on the Medical Dictionary for Regulatory Activities version 27.0 (International Council for Harmonisation/ MedDRA Maintenance and Support Services Organization, McLean, VA) [18]. For case extraction, we focused on PT for EM (PT code: 10015218).

Time-to-onset analysis

**Figure 1:**
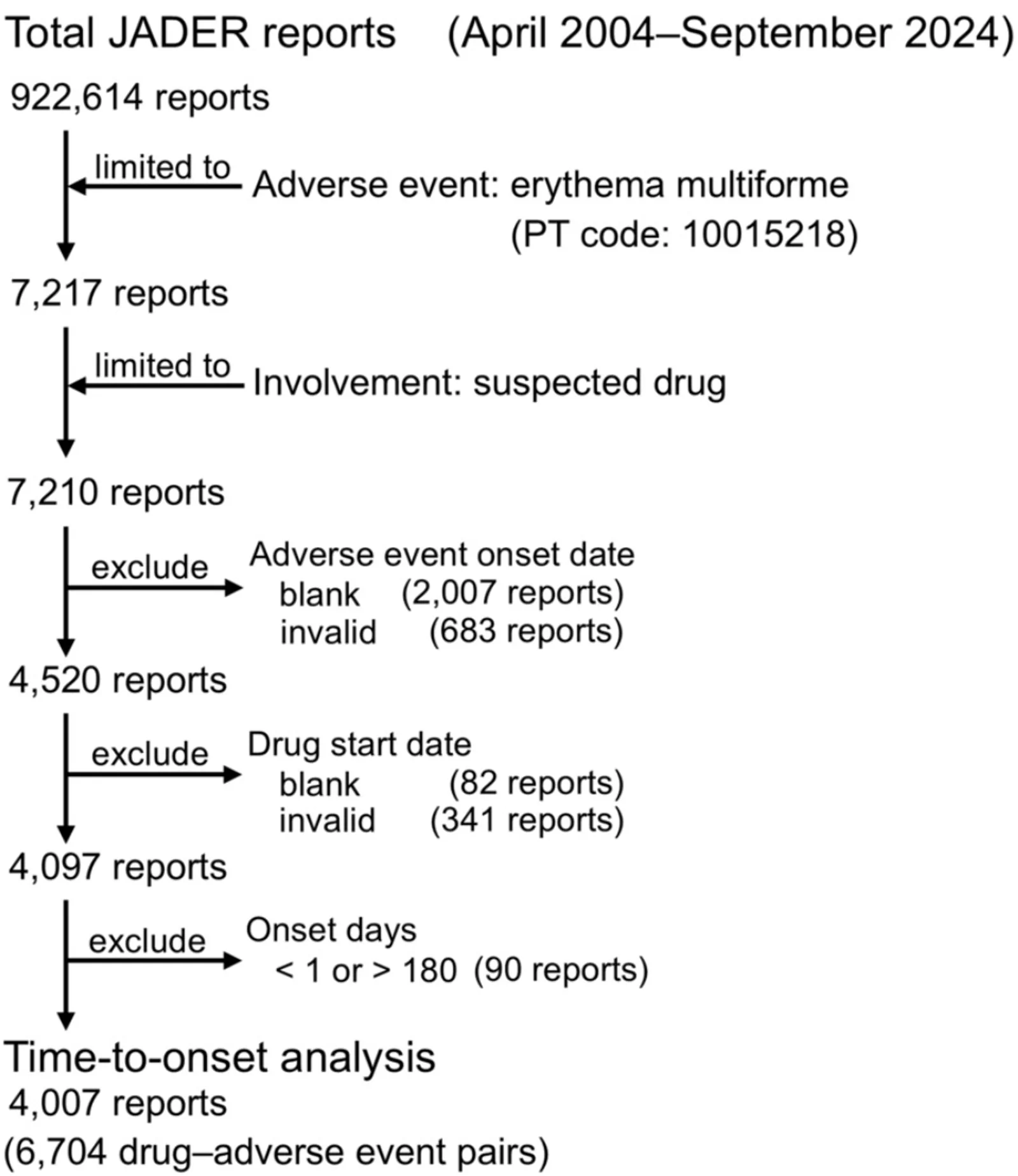
Flow diagram of report selection for the time-to-onset analysis of erythema multiforme. The final dataset included 4,007 unique reports and 6,704 suspected drug-adverse event pairs JADER: Japanese Adverse Drug Event Report; PT: preferred term

One record was retained for each combination of report identification number and generic suspected drug name. Complete eight-digit drug administration start dates and AE onset dates were required. Time to onset was calculated as the AE onset date minus the drug administration start date plus one day and was restricted to 1-180 days. Because a single EM report may list multiple suspected drugs, the unit of analysis in this study was the suspected drug-AE pair rather than the unique report ID. Accordingly, the 4,007 unique EM reports yielded a total of 6,704 suspected drug-AE pairs, which constituted the analytical dataset for the time-to-onset analyses (Figure 1). All subsequent analyses, including the age-related rank correlation analysis and Kaplan-Meier analysis described below, were performed at the level of drug-AE pairs.

Age-related rank correlation analysis

For the rank correlation analysis, drug-AE pairs were categorized into the following age groups: <10, 10-19, 20-29, 30-39, 40-49, 50-59, 60-69, 70-79, and ≥80 years. Age entries that could not be mapped to the predefined 10-year categories, including reporter-defined age groups (e.g., elderly, child, adult) and missing or unknown age information, were categorized as "Unknown" in descriptive summaries but excluded from the rank correlation and age-group Kaplan-Meier analyses. The association between ordered age categories and reported time to onset was evaluated using Kendall’s tau-b rank correlation analysis. Age groups were assigned ordinal scores in ascending order, and the analysis was performed in Python (version 3.12.3; Python Software Foundation, Beaverton, OR) using the kendalltau function with the b variant in SciPy (version 1.13.1; SciPy Community, Austin, TX) [19].

Kaplan-Meier analysis

For descriptive visualization, cumulative time-to-onset curves were generated for the nine age groups overall and separately for men and women. The same approach was applied to reports with a single suspected drug. The number of suspected drugs per report was determined from the original drug table before exclusions based on date completeness or the 1-180-day time-to-onset window. For an exploratory comparison, the drug-AE pairs were categorized according to age group (<60 and ≥60 years). The cutoff of 60 years was selected pragmatically from the available 10-year age categories for an exploratory comparison and was not intended to represent an established clinical threshold. The time-to-onset profiles of EM were compared between the stratified age groups using the Kaplan-Meier method with a log-rank test. To investigate the influence of drug type on EM onset, suspected drugs were categorized using the ATC classification system (first level). We analyzed time to onset for each ATC group and performed a stratified analysis by age group. The ATC-stratified analyses were exploratory; no adjustment for multiple comparisons (e.g., the Bonferroni method, Holm method, or false discovery rate control) was applied, and the p values were interpreted as nominal. The lifelines library (version 0.30.0) in Python was used for the analysis [20].

## Results

After applying the eligibility criteria for the time-to-onset analysis, 4,007 unique EM reports were included, yielding 6,704 suspected drug-AE pairs (Figure 1). Among these reports, 1,738 involved male patients (43.4%), 2,249 involved female patients (56.1%), and 20 had unknown sex. Because a single report ID could include multiple suspected drugs, the time-to-onset analysis was based on suspected drug-AE pairs rather than unique report IDs. The overall median time to onset was 10.0 days (interquartile range: 5.0-17.0). Although the median time to onset was 10.0 days for both sexes, the Kaplan-Meier curves showed a statistically significant difference (log-rank test, p = 0.012; Table 1).

**Table 1:** Time to onset of erythema multiforme by sex and age with Kaplan-Meier analysis ^a^Counts represent suspected drug-adverse event pairs; a single report ID may correspond to multiple pairs involving different suspected drugs ^b^Log-rank test comparing survival curves between male and female individuals ^c^Log-rank test comparing survival curves between individuals aged <60 and ≥60 IQR: interquartile range

Sex	Age	Counts^a^	Median (IQR)	p value
All	-	6,704	10.0 (5.0-17.0)	-
Male	-	2,967	10.0 (5.0-20.0)	0.012^b^
Female	-	3,708	10.0 (5.0-16.0)	
All	<60	3,080	8.0 (3.0-13.0)	<0.001^c^
	≥60	3,551	12.0 (7.0-24.5)	
Male	<60	1,341	8.0 (3.0-13.0)	<0.001^c^
	≥60	1,590	12.0 (7.0-28.0)	
Female	<60	1,729	9.0 (4.0-13.0)	<0.001^c^
	≥60	1,952	11.0 (7.0-22.0)	

**Figure 2:**
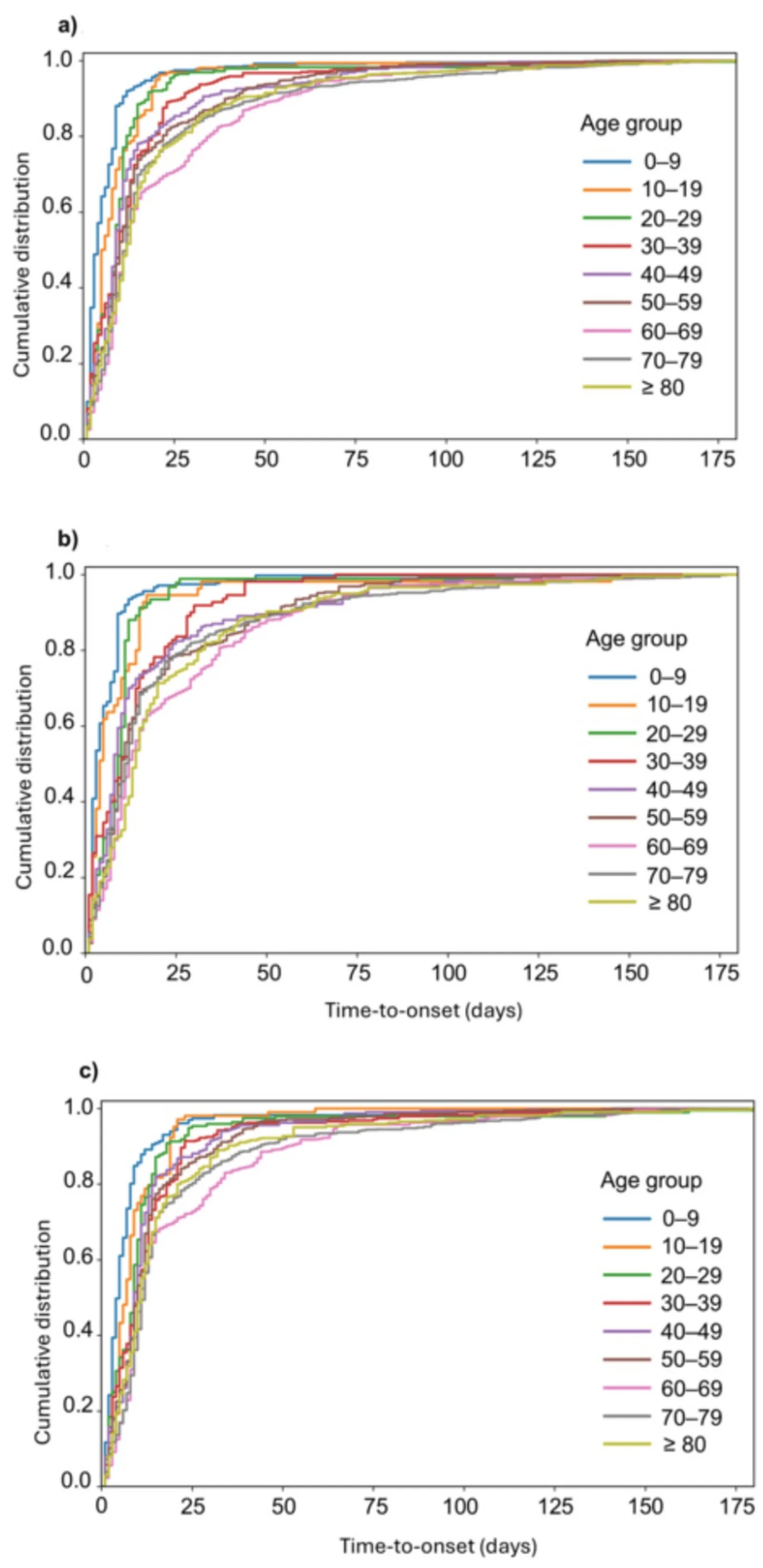
Cumulative distributions of reported time to onset of erythema multiforme by age group. Curves are shown for (a) all drug-adverse event pairs, (b) male patients, and (c) female patients Median time to onset and interquartile ranges are presented in Table 2

The reported time to onset showed a positive association with ordered age category, ranging from a median of 4.0 days in the 0-9-year age group to 12.0 days in the ≥80-year age group (Kendall’s tau-b = 0.188, p < 0.001; Table 2, Figure 2). Similar positive associations were observed in males (Kendall’s tau-b = 0.214, p < 0.001) and female patients (Kendall’s tau-b = 0.159, p < 0.001).

**Table 2:** Time to onset of erythema multiforme by sex and age with Kendall’s tau-b rank correlation analysis ^a^Counts represent suspected drug-adverse event pairs; a single report ID may correspond to multiple pairs involving different suspected drugs ^b^p value from Kendall’s tau-b rank correlation test IQR: interquartile range

Sex	Age	Counts^a^	Median (IQR)	Kendall's tau-b	p value^b^
All	0-9	625	4.0 (2.0-8.0)	0.188	<0.001
	10-19	160	5.5 (4.0-11.0)		
	20-29	288	9.0 (4.0-11.0)		
	30-39	457	10.0 (3.0-15.0)		
	40-49	603	9.0 (6.0-14.0)		
	50-59	947	10.0 (6.0-17.0)		
	60-69	1,605	12.0 (8.0-30.0)		
	70-79	1,359	12.0 (7.0-21.0)		
	≥80	587	12.0 (6.0-21.0)		
Male	0-9	387	3.0 (2.0-9.0)	0.214	<0.001
	10-19	55	4.0 (2.5-11.5)		
	20-29	92	9.0 (4.8-11.0)		
	30-39	110	10.0 (2.0-17.5)		
	40-49	284	8.0 (5.0-18.2)		
	50-59	413	10.0 (7.0-22.0)		
	60-69	727	12.0 (8.0-32.0)		
	70-79	626	11.0 (7.0-21.0)		
	≥80	237	14.0 (7.0-27.0)		
Female	0-9	231	5.0 (3.0-8.0)	0.159	<0.001
	10-19	104	7.0 (5.0-10.2)		
	20-29	196	9.0 (3.8-12.0)		
	30-39	347	10.0 (4.0-15.0)		
	40-49	319	9.0 (6.0-13.0)		
	50-59	532	10.0 (5.0-14.0)		
	60-69	875	11.0 (8.0-28.0)		
	70-79	731	12.0 (8.0-19.0)		
	≥80	346	11.0 (6.0-17.8)		

**Figure 3:**
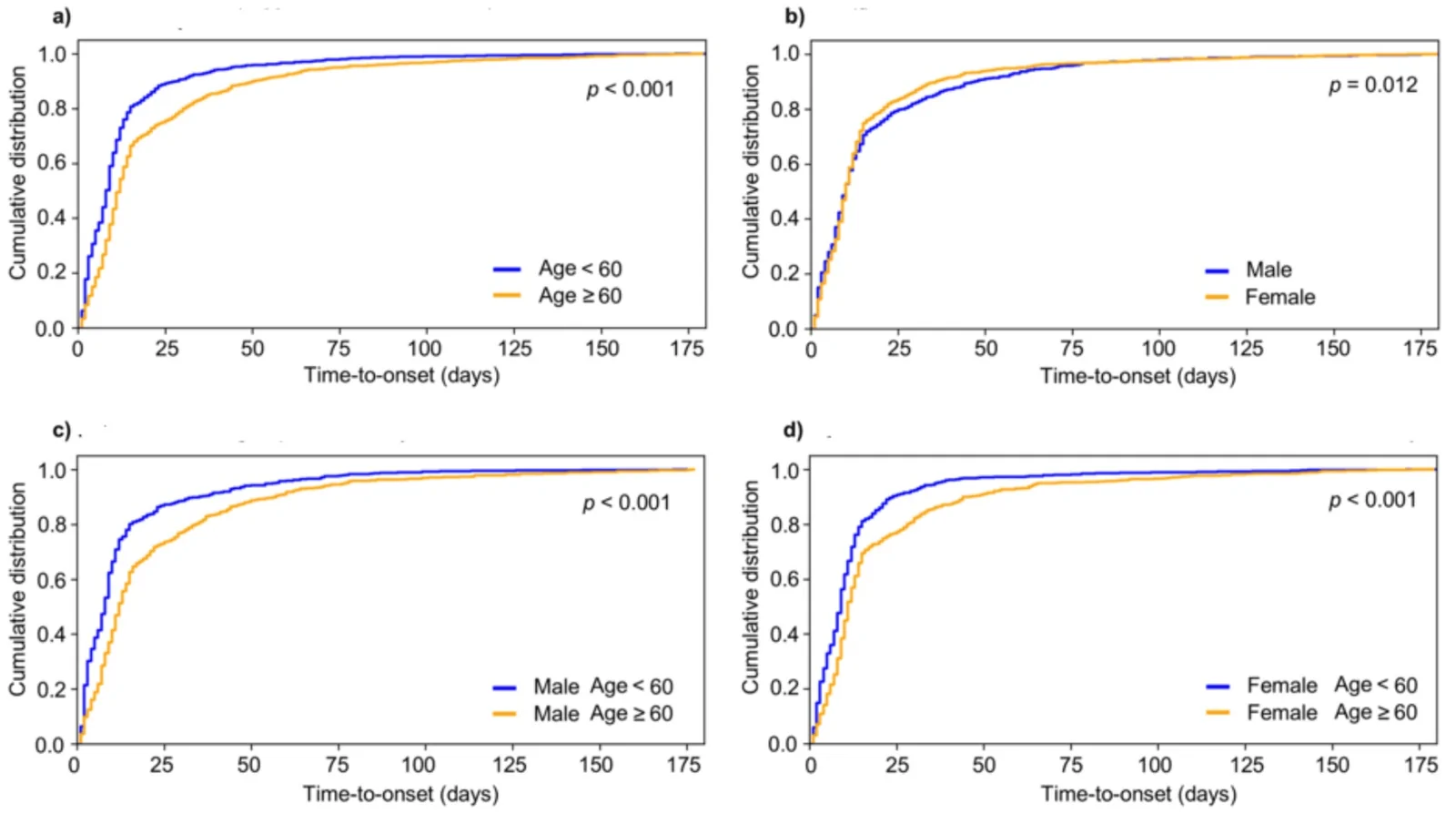
Cumulative distributions of time to onset of erythema multiforme estimated using the Kaplan-Meier method. (a) Stratified by age group (<60 and ≥60 years). (b) Stratified by sex (male and female). (c) For the male subgroup, stratified by age group (<60 and ≥60 years). (d) For the female subgroup, stratified by age group (<60 and ≥60 years)

In an exploratory comparison, the median time to onset was significantly shorter in individuals aged <60 years (8.0 days) than in those aged ≥60 years (12.0 days) (log-rank test, p < 0.001; Table 1, Figure 3).

**Figure 4:**
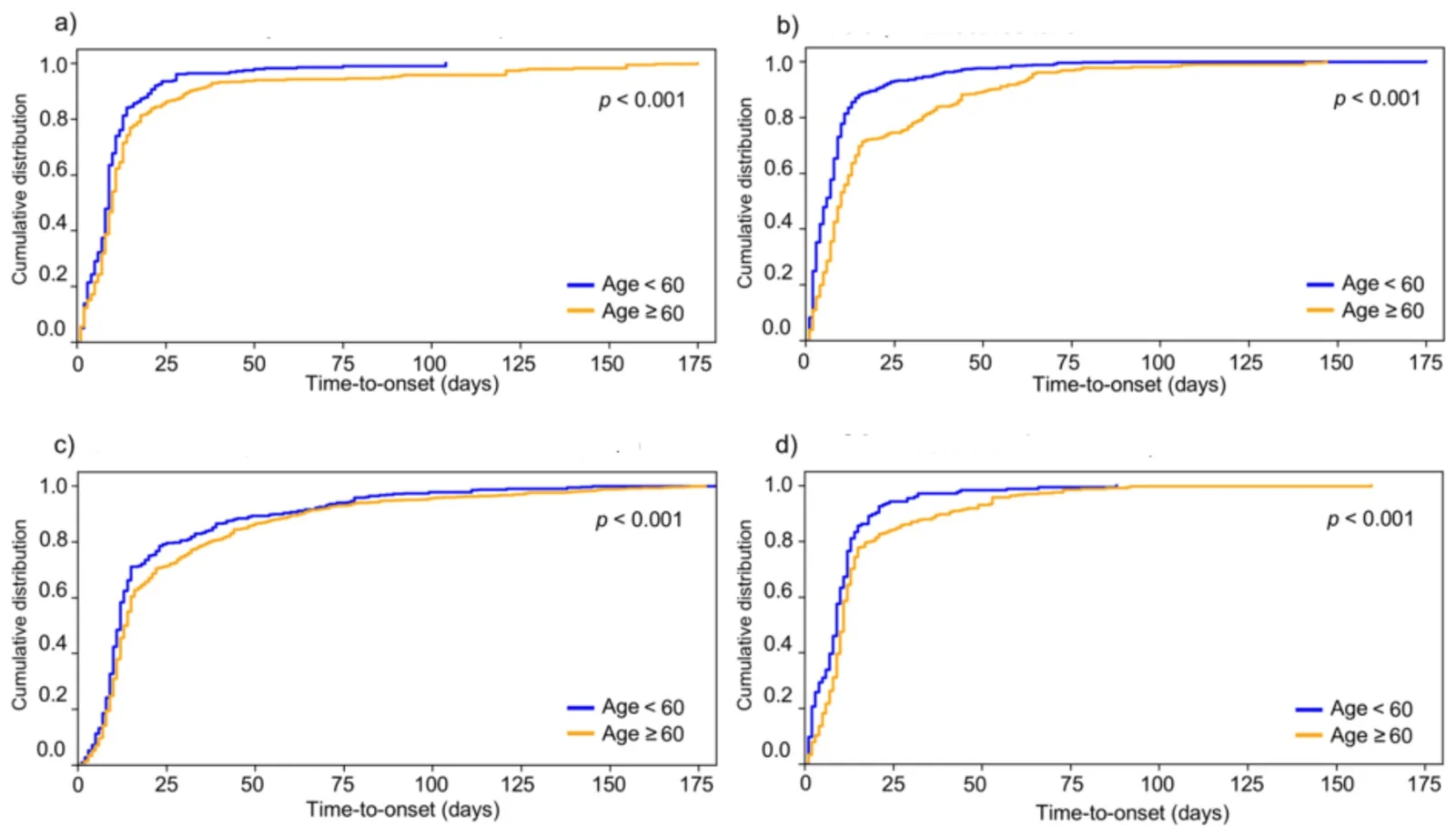
Cumulative distributions of time to onset of erythema multiforme estimated using the Kaplan-Meier method by age group (<60 and ≥60 years) within four ATC classes. (a) Alimentary tract and metabolism (ATC A). (b) Anti-infectives for systemic use (ATC J). (c) Antineoplastic and immunomodulating agents (ATC L). (d) Musculoskeletal system (ATC M) ATC: Anatomical Therapeutic Chemical

To further investigate the influence of drug type, we performed a stratified analysis based on the ATC classification. In ATC categories A, J, L, and M, time to onset was significantly longer in individuals aged ≥60 years than in those aged <60 years (log-rank test, all p < 0.001; Table 3). The corresponding Kaplan-Meier curves are shown in Figure 4.

**Table 3:** Time to onset of erythema multiforme by ATC first-level classification and age group ^a^Counts represent suspected drug-adverse event pairs; a single report ID may correspond to multiple pairs involving different suspected drugs ^b^The chi-square statistic and p values were calculated using the log-rank test ATC: Anatomical Therapeutic Chemical; IQR: interquartile range; Min: minimum; Max: maximum

ATC	Category	Age	Counts^a^	Min	Median (IQR)	Max	Chi-square^b^	p value^b^
A	Alimentary tract and metabolism	<60	276	1.0	9.0 (5.0-12.0)	104.0	16.071	<0.001
		≥60	333	1.0	10.0 (7.0-15.0)	175.0		
B	Blood and blood forming organs	<60	51	1.0	9.0 (3.0-12.0)	84.0	8.742	0.003
		≥60	102	1.0	12.0 (8.3-24.8)	145.0		
C	Cardiovascular system	<60	48	2.0	10.5 (7.0-21.0)	131.0	4.012	0.045
		≥60	165	1.0	14.0 (7.0-28.0)	175.0		
D	Dermatologicals	<60	37	1.0	10.0 (7.0-16.0)	47.0	6.006	0.014
		≥60	46	1.0	16.0 (10.0-28.0)	127.0		
G	Genitourinary system and sex hormones	<60	17	2.0	12.0 (7.0-16.0)	118.0	0.001	0.971
		≥60	20	2.0	18.0 (9.8-25.3)	89.0		
H	Systemic hormonal preparations, excluding sex hormones and insulins	<60	16	1.0	12.5 (3.8-17.3)	32.0	2.111	0.146
		≥60	25	1.0	14.0 (7.0-35.0)	99.0		
J	Anti-infectives for systemic use	<60	1189	1.0	6.0 (3.0-10.0)	175.0	158.332	<0.001
		≥60	720	1.0	10.0 (6.0-27.0)	147.0		
L	Antineoplastic and immunomodulating agents	<60	444	1.0	12.0 (9.0-20.3)	180.0	12.960	<0.001
		≥60	1142	1.0	13.5 (10.0-30.0)	177.0		
M	Musculoskeletal system	<60	174	1.0	9.0 (3.0-12.0)	88.0	17.451	<0.001
		≥60	328	1.0	11.0 (7.0-15.0)	160.0		
N	Nervous system	<60	343	1.0	13.0 (7.0-23.0)	171.0	2.854	0.091
		≥60	254	1.0	15.0 (5.0-30.0)	115.0		
P	Antiparasitic products, insecticides, and repellents	<60	13	2.0	15.0 (13.0-18.0)	22.0	2.356	0.125
		≥60	7	3.0	5.0 (4.0-11.5)	21.0		
R	Respiratory system	<60	174	1.0	7.0 (3.0-9.0)	60.0	9.356	0.002
		≥60	67	1.0	8.0 (4.0-16.0)	127.0		
S	Sensory organs	<60	2	5.0	5.0 (5.0-5.0)	5.0	2.000	0.157
		≥60	1	6.0	6.0 (6.0-6.0)	6.0		
V	Various	<60	6	2.0	8.0 (5.0-15.5)	92.0	0.076	0.782
		≥60	30	1.0	8.0 (6.0-10.0)	110.0		
-	Others (non-ATC classified drugs)	<60	290	1.0	5.0 (2.0-12.0)	162.0	9.736	0.002
		≥60	311	1.0	9.0 (4.0-15.0)	179.0		

The distributions of suspected drug-AE pairs across ATC first-level categories by age group (Table 4) and sex (Table 5) are shown. The most frequently reported drug classes were anti-infectives for systemic use (ATC code J, 1,922 drug-AE pairs) and antineoplastic and immunomodulating agents (ATC code L, 1,621 drug-AE pairs).

**Table 4:** Distribution of suspected drug-adverse event pairs associated with erythema multiforme by age group according to the ATC first-level classification ATC: Anatomical Therapeutic Chemical

ATC code	Category	Total	0-9 (%)	10-19 (%)	20-29 (%)	30-39 (%)	40-49 (%)	50-59 (%)	60-69 (%)	70-79 (%)	≥80 (%)	Unknown (%)
Total	-	6,704	625 (9.3)	160 (2.4)	288 (4.3)	457 (6.8)	603 (9.0)	947 (14.1)	1,605 (23.9)	1,359 (20.3)	587 (8.8)	73 (1.1)
A	Alimentary tract and metabolism	616	23 (3.7)	14 (2.3)	21 (3.4)	52 (8.4)	69 (11.2)	97 (15.7)	137 (22.2)	136 (22.1)	60 (9.7)	7 (1.1)
B	Blood and blood forming organs	154	13 (8.4)	2 (1.3)	5 (3.2)	2 (1.3)	11 (7.1)	18 (11.7)	30 (19.5)	44 (28.6)	28 (18.2)	1 (0.6)
C	Cardiovascular system	213	0 (0.0)	0 (0.0)	3 (1.4)	4 (1.9)	16 (7.5)	25 (11.7)	52 (24.4)	72 (33.8)	41 (19.2)	0 (0.0)
D	Dermatologicals	84	14 (16.7)	2 (2.4)	2 (2.4)	5 (6.0)	4 (4.8)	10 (11.9)	25 (29.8)	12 (14.3)	9 (10.7)	1 (1.2)
G	Genitourinary system and sex hormones	37	0 (0.0)	0 (0.0)	3 (8.1)	10 (27.0)	4 (10.8)	0 (0.0)	6 (16.2)	6 (16.2)	8 (21.6)	0 (0.0)
H	Systemic hormonal preparations, excluding sex hormones and insulins	41	1 (2.4)	0 (0.0)	2 (4.9)	2 (4.9)	5 (12.2)	6 (14.6)	14 (34.1)	7 (17.1)	4 (9.8)	0 (0.0)
J	Anti-infectives for systemic use	1,922	440 (22.9)	73 (3.8)	94 (4.9)	139 (7.2)	183 (9.5)	260 (13.5)	379 (19.7)	234 (12.2)	107 (5.6)	13 (0.7)
L	Antineoplastic and immunomodulating agents	1,621	2 (0.1)	10 (0.6)	14 (0.9)	43 (2.7)	121 (7.5)	254 (15.7)	566 (34.9)	475 (29.3)	101 (6.2)	35 (2.2)
M	Musculoskeletal system	505	2 (0.4)	4 (0.8)	29 (5.7)	39 (7.7)	40 (7.9)	60 (11.9)	116 (23.0)	121 (24.0)	91 (18.0)	3 (0.6)
N	Nervous system	598	34 (5.7)	33 (5.5)	50 (8.4)	81 (13.5)	55 (9.2)	90 (15.1)	104 (17.4)	103 (17.2)	47 (7.9)	1 (0.2)
P	Antiparasitic products, insecticides, and repellents	20	0 (0.0)	0 (0.0)	3 (15.0)	4 (20.0)	6 (30.0)	0 (0.0)	4 (20.0)	1 (5.0)	2 (10.0)	0 (0.0)
R	Respiratory system	245	85 (34.7)	6 (2.4)	20 (8.2)	28 (11.4)	23 (9.4)	12 (4.9)	31 (12.7)	19 (7.8)	17 (6.9)	4 (1.6)
S	Sensory organs	3	0 (0.0)	0 (0.0)	2 (66.7)	0 (0.0)	0 (0.0)	0 (0.0)	0 (0.0)	1 (33.3)	0 (0.0)	0 (0.0)
V	Various	38	0 (0.0)	0 (0.0)	0 (0.0)	0 (0.0)	1 (2.6)	5 (13.2)	16 (42.1)	9 (23.7)	5 (13.2)	2 (5.3)
-	Others (non-ATC classified drugs)	607	11 (1.8)	16 (2.6)	40 (6.6)	48 (7.9)	65 (10.7)	110 (18.1)	125 (20.6)	119 (19.6)	67 (11.0)	6 (1.0)

**Table 5:** Distribution of suspected drug-adverse event pairs associated with erythema multiforme by sex according to the ATC first-level classification ATC: Anatomical Therapeutic Chemical

ATC code	Category	Total	Male (%)	Female (%)	Unknown (%)
Total	-	6,704	2,967 (44.3)	3,708 (55.3)	29 (0.4)
A	Alimentary tract and metabolism	616	233 (37.8)	383 (62.2)	0 (0.0)
B	Blood and blood forming organs	154	70 (45.5)	84 (54.5)	0 (0.0)
C	Cardiovascular system	213	88 (41.3)	125 (58.7)	0 (0.0)
D	Dermatologicals	84	35 (41.7)	46 (54.8)	3 (3.6)
G	Genitourinary system and sex hormones	37	14 (37.8)	23 (62.2)	0 (0.0)
H	Systemic hormonal preparations, excluding sex hormones and insulins	41	22 (53.7)	19 (46.3)	0 (0.0)
J	Anti-infectives for systemic use	1,922	880 (45.8)	1,033 (53.7)	9 (0.5)
L	Antineoplastic and immunomodulating agents	1,621	822 (50.7)	787 (48.6)	12 (0.7)
M	Musculoskeletal system	505	179 (35.4)	326 (64.6)	0 (0.0)
N	Nervous system	598	186 (31.1)	412 (68.9)	0 (0.0)
P	Antiparasitic products, insecticides, and repellents	20	3 (15.0)	17 (85.0)	0 (0.0)
R	Respiratory system	245	132 (53.9)	110 (44.9)	3 (1.2)
S	Sensory organs	3	1 (33.3)	2 (66.7)	0 (0.0)
V	Various	38	21 (55.3)	17 (44.7)	0 (0.0)
-	Others (non-ATC classified drugs)	607	281 (46.3)	324 (53.4)	2 (0.3)

The number of suspected drugs per report was also summarized by age group (Table 6), with the highest number of EM reports observed in the 60-69-year age group (n = 981).

**Table 6:** Distribution of the number of suspected drugs per report by age group in all reports and erythema multiforme reports EM: erythema multiforme

Reports	Age group	Total	1 drug (%)	2 drugs (%)	3 drugs (%)	4 drugs (%)	5 drugs (%)	≥6 drugs (%)
All	Total	368,639	180,119 (48.9)	79,613 (21.6)	39,218 (10.6)	22,959 (6.2)	12,587 (3.4)	34,143 (9.3)
	0-9	14,674	7,503 (51.1)	2,672 (18.2)	1,658 (11.3)	1,157 (7.9)	641 (4.4)	1,043 (7.1)
	10-19	10,106	5,683 (56.2)	1,702 (16.8)	1,038 (10.3)	496 (4.9)	316 (3.1)	871 (8.6)
	20-29	11,878	6,161 (51.9)	2,037 (17.1)	1,236 (10.4)	694 (5.8)	460 (3.9)	1,290 (10.9)
	30-39	18,897	9,018 (47.7)	3,709 (19.6)	1,992 (10.5)	1,259 (6.7)	738 (3.9)	2,181 (11.5)
	40-49	26,776	12,646 (47.2)	5,435 (20.3)	2,899 (10.8)	1,791 (6.7)	1,067 (4.0)	2,938 (11.0)
	50-59	46,937	20,471 (43.6)	10,022 (21.4)	5,369 (11.4)	3,406 (7.3)	1,887 (4.0)	5,782 (12.3)
	60-69	84,485	37,232 (44.1)	18,812 (22.3)	9,580 (11.3)	5,754 (6.8)	3,263 (3.9)	9,844 (11.7)
	70-79	96,333	47,164 (49.0)	22,620 (23.5)	10,187 (10.6)	5,814 (6.0)	2,948 (3.1)	7,600 (7.9)
	≥80	51,498	29,550 (57.4)	11,319 (22.0)	4,770 (9.3)	2,344 (4.6)	1,144 (2.2)	2,371 (4.6)
	Unknown	7,055	4,691 (66.5)	1,285 (18.2)	489 (6.9)	244 (3.5)	123 (1.7)	223 (3.2)
EM cases	Total	4,007	1,916 (47.8)	901 (22.5)	522 (13.0)	255 (6.4)	145 (3.6)	268 (6.7)
	0-9	303	146 (48.2)	73 (24.1)	24 (7.9)	28 (9.2)	10 (3.3)	22 (7.3)
	10-19	92	56 (60.9)	11 (12.0)	5 (5.4)	7 (7.6)	5 (5.4)	8 (8.7)
	20-29	163	73 (44.8)	37 (22.7)	26 (16.0)	10 (6.1)	10 (6.1)	7 (4.3)
	30-39	256	124 (48.4)	62 (24.2)	31 (12.1)	14 (5.5)	3 (1.2)	22 (8.6)
	40-49	357	163 (45.7)	85 (23.8)	49 (13.7)	20 (5.6)	20 (5.6)	20 (5.6)
	50-59	536	239 (44.6)	99 (18.5)	80 (14.9)	50 (9.3)	21 (3.9)	47 (8.8)
	60-69	981	442 (45.1)	220 (22.4)	135 (13.8)	59 (6.0)	46 (4.7)	79 (8.1)
	70-79	870	417 (47.9)	222 (25.5)	113 (13.0)	50 (5.7)	23 (2.6)	45 (5.2)
	≥80	395	218 (55.2)	85 (21.5)	55 (13.9)	15 (3.8)	6 (1.5)	16 (4.1)
	Unknown	54	38 (70.4)	7 (13.0)	4 (7.4)	2 (3.7)	1 (1.9)	2 (3.7)

**Figure 5:**
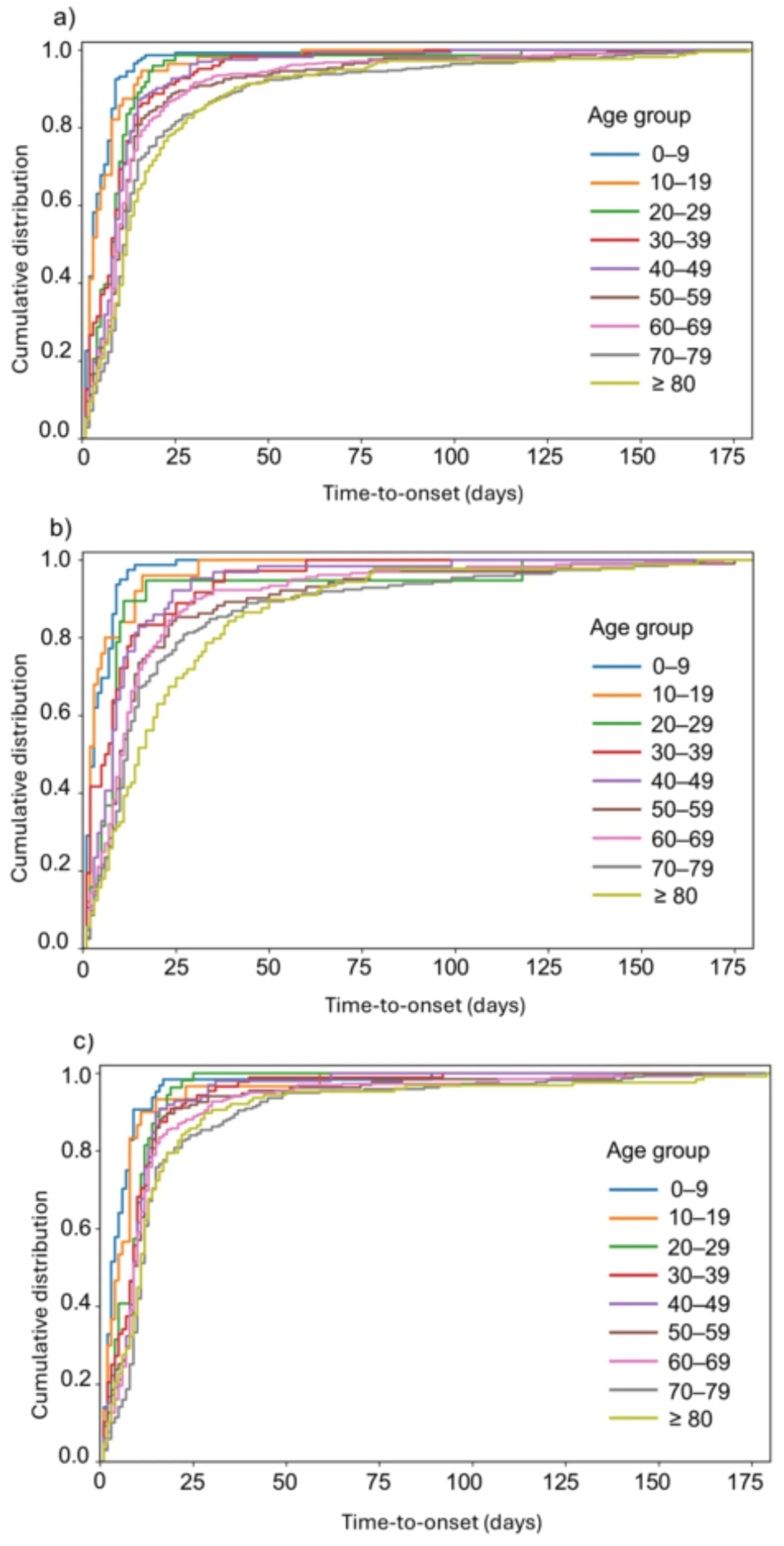
Cumulative distributions of reported time to onset of erythema multiforme by age group among reports with a single suspected drug. Curves are shown for (a) all reports, (b) male patients, and (c) female patients Median time to onset and interquartile ranges are presented in Table 7

To assess the robustness of the findings and evaluate the potential influence of reports involving multiple suspected drugs, we conducted an additional analysis restricted to reports with a single suspected drug (Figure 5, Table 7). Consistent with the overall analysis, older age was associated with longer reported time to onset. Kendall’s tau-b rank correlation analysis showed a significant positive association between ordered age category and reported time to onset (Kendall’s tau-b = 0.237, p < 0.001).

**Table 7:** Time to onset of erythema multiforme by age group among reports with a single suspected drug ^a^Counts represent reports with a single suspected drug ^b^p value from Kendall’s tau-b rank correlation test IQR: interquartile range

Sex	Age	Counts^a^	Median (IQR)	Kendall's tau-b	p value^b^
All	0-9	146	3.0 (2.0-7.0)	0.237	<0.001
	10-19	56	3.0 (2.0-8.0)		
	20-29	73	8.0 (4.0-11.0)		
	30-39	124	8.0 (2.0-12.0)		
	40-49	163	9.0 (5.0-12.0)		
	50-59	239	10.0 (7.0-14.0)		
	60-69	442	10.0 (6.0-15.0)		
	70-79	417	12.0 (8.0-18.0)		
	≥80	218	12.0 (7.0-21.8)		
	Unknown	38	10.5 (4.5-15.0)		
Male	0-9	79	3.0 (1.0-7.0)	0.276	<0.001
	10-19	25	2.0 (2.0-5.0)		
	20-29	19	8.0 (5.0-9.0)		
	30-39	36	6.5 (2.0-12.0)		
	40-49	64	8.0 (4.0-11.2)		
	50-59	102	10.0 (7.0-16.8)		
	60-69	181	11.0 (6.0-17.0)		
	70-79	198	12.0 (7.0-22.0)		
	≥80	89	15.0 (7.0-31.0)		
	Unknown	18	12.0 (7.2-20.2)		
Female	0-9	64	3.0 (2.0-7.2)	0.197	<0.001
	10-19	30	5.0 (2.0-8.0)		
	20-29	54	9.0 (4.0-11.8)		
	30-39	88	9.0 (3.8-12.0)		
	40-49	99	9.0 (6.0-12.0)		
	50-59	135	9.0 (5.5-13.0)		
	60-69	261	9.0 (7.0-13.0)		
	70-79	219	11.0 (8.5-15.0)		
	≥80	127	11.0 (6.0-16.5)		
	Unknown	13	9.0 (6.0-14.0)		

## Discussion

To the best of our knowledge, this is the first study to analyze the time to onset of drug-associated EM by age group using data from the JADER database. The findings indicated an association between older age and longer reported time to onset of EM.

A potential confounding factor in analyzing age-related trends is the difference in prescribed drug classes. Our data indicated distinct reporting profiles between age groups. Pediatric cases were predominantly associated with anti-infectives for systemic use (ATC code J) and the respiratory system (ATC code R), whereas elderly cases were more frequently associated with blood and blood-forming organs (ATC code B), the cardiovascular system (ATC code C), antineoplastic and immunomodulating agents (ATC code L), and the musculoskeletal system (ATC code M). However, the stratified analysis showed that the reported time to onset was longer in individuals aged ≥60 years than in those aged <60 years across major ATC categories, excluding those with limited report numbers. This pattern was observed in various categories, including anti-infectives, and was not limited to immunomodulating agents. Notably, the number of suspected drugs per report was not higher in older age groups (Table 6). In addition, an association between older age and longer reported time to onset was observed in the analysis restricted to reports with a single suspected drug (Figure 5, Table 7). These findings suggest that the observed association is unlikely to be explained solely by differences in suspected drug types or by reports involving multiple suspected drugs, although unmeasured patient-related factors may also have contributed.

We also evaluated sex-related differences in reported time to onset. Although the Kaplan-Meier analysis showed a significant difference between men and women, the median time to onset was 10.0 days in both sexes. This indicates that the statistical significance was driven by the overall distribution pattern rather than the median, which may be partly attributable to sex-based differences in suspected drugs. Our analysis indicated that female cases were more frequently associated with nervous system and musculoskeletal system agents, whereas male cases showed a higher proportion of reports involving respiratory system drugs and systemic hormonal preparations.

Findings from a spontaneous reporting database cannot establish causal biological mechanisms. Nevertheless, because EM is considered an immune-mediated condition, age-related immune changes may provide a hypothesis-generating context for the observed association. Previous studies have described age-related differences in AE profiles and immune responses in older adults [8,9], and immunosenescence has been shown to alter T-cell function [7]. However, the contribution of immunosenescence to the observed association between age and reported time to onset of EM could not be directly evaluated in the present study.

Sex-related immune differences may also provide a hypothesis-generating context for interpreting the observed differences in onset profiles. Women generally have stronger immune responses to infections and vaccinations than men [10], and sex differences in the susceptibility and severity of viral infections have been attributed, at least in part, to the influence of sex hormones on immune cell function [10,11]. Estrogen receptors are widely expressed in cells of the immune system and may influence both innate and adaptive immune responses [21,22]. However, the present study could not evaluate hormonal status, estrogen receptor expression, or other immunological mechanisms. Therefore, the possible contribution of estrogen-related immune modulation should be interpreted as a hypothesis rather than a demonstrated mechanism. These sex-related differences should nevertheless be interpreted cautiously because the median time to onset was identical between men and women.

Our findings from the JADER database indicated an association between older age and longer reported time to onset of EM, whereas sex-related differences were observed in the overall distribution of reported time to onset despite identical median values. Further mechanistic or clinical studies, including measurement of key immune mediators in patient samples, are warranted. Regarding the clinical interpretation of these findings, the median reported time to onset differed by four days between individuals aged <60 years and those aged ≥60 years. Although this difference was statistically significant, the absolute difference was modest, and its clinical relevance in routine pharmacovigilance or patient monitoring remains uncertain. Both median values were within the first two weeks after drug initiation. However, the interquartile range was wider in individuals aged ≥60 years than in those aged <60 years (7.0-24.5 vs. 3.0-13.0 days), indicating greater dispersion and a later upper quartile in the older group. Therefore, these findings may raise awareness of the possibility of later reported onset in older individuals but do not support specific age-based monitoring recommendations. Prospective studies are needed to determine the clinical relevance of this association.

Limitations

This study has inherent limitations related to spontaneous reporting systems, such as overreporting, underreporting, and missing data. In particular, reporting biases, such as the underreporting of nonfatal events, may skew the demographic distribution [23]. However, we primarily focused on time to onset rather than incidence, and the consistency of the age-related trends across the stratified analyses supports the interpretation of the observed age-related pattern. The 1-180-day observation window was selected with reference to previous JADER-based time-to-onset studies that either used a 180-day cutoff or evaluated AE onset within 180 days in a secondary analysis [24,25]. However, no standardized statistical criterion or established consensus exists regarding the optimal upper limit. Additional analyses using 90- and 120-day upper limits showed the same age-related pattern as the primary 180-day analysis (i.e., longer reported time to onset in older individuals), although the number of included drug-AE pairs varied depending on the selected upper limit (data not shown). For both alternative observation windows, the overall median reported time to onset of EM was 10.0 days; the corresponding medians were 8.0 days in individuals aged <60 years and 11.0 days in those aged ≥60 years (log-rank test, p < 0.001 for both analyses). Kendall’s tau-b values were 0.181 and 0.184 for the 90- and 120-day analyses, respectively (p < 0.001 for both analyses).

Another limitation is that our analysis was restricted to drug-associated EM cases reported in the JADER database. Therefore, the findings regarding age-related onset patterns cannot be directly extrapolated to EM caused by infections, autoimmune diseases, or other nondrug-related etiologies. The JADER database comprises reports submitted in Japan. Because drug availability, prescribing practices, healthcare systems, and reporting behavior may differ across countries and reporting systems, the generalizability of these findings to other populations and data sources may be limited. External factors, such as safety communications and market dynamics, may also influence reporting patterns. The JADER database does not provide sufficiently complete and consistently recorded information to adjust for potentially relevant confounders, such as comorbidities, concomitant medications, treatment indication, drug dose, and clinical severity. Consequently, potential confounding by unmeasured or incompletely recorded patient- and treatment-related factors cannot be excluded.

From an analytical perspective, each suspected drug-AE pair was treated as an independent observation in our time-to-onset analyses. Consequently, reports containing multiple suspected drugs contributed multiple observations and may have had greater influence on the analysis than reports containing a single suspected drug. In addition, multiple pairs originating from the same report may share within-report correlations, which were not explicitly modeled. This nonindependence may lead to underestimation of statistical uncertainty and should be considered when interpreting the p values. Accordingly, the findings should be interpreted as reporting patterns at the drug-AE pair level rather than as patient-level estimates. Nevertheless, the number of suspected drugs per report was not higher in older age groups (Table 6), and a similar age-related pattern was observed when the analysis was restricted to reports with a single suspected drug (Figure 5, Table 7). These findings suggest that the main result was not explained solely by the inclusion of reports containing multiple suspected drugs, although residual within-report dependence cannot be excluded. Because no adjustment for multiple comparisons was applied to the exploratory ATC-stratified analyses, the possibility of type I error should be considered when interpreting the category-specific findings.

To better understand the clinical relevance and potential causal relationships underlying these findings, future research involving electronic medical records and claims databases is essential. In addition, future research using more granular data, including detailed patient demographics, clinical conditions, and environmental factors, is required to elucidate the mechanisms underlying these findings. Furthermore, examining whether age and sex influence the onset of other immune-related drug-induced AEs beyond EM could provide additional insights.

## Conclusions

Our analysis identified an association between older age and longer reported time to onset of EM in the JADER database. The median time to onset was 10.0 days in both men and women. However, Kaplan-Meier curves suggested differences in the distribution of reported onset profiles by sex. These findings suggest age-related differences and possible sex-related differences in the distribution of reported time to onset of EM. Although causality cannot be inferred from a spontaneous reporting database, the findings may raise awareness of the possibility of later reported onset of EM in older patients. Prospective studies are needed to confirm the clinical relevance of these findings.

## References

[1] Soares A, Sokumbi O (2021). Recent updates in the treatment of erythema multiforme. Medicina (Kaunas).

[2] Lamoreux MR, Sternbach MR, Hsu WT (2006). Erythema multiforme. Am Fam Physician.

[3] Trayes KP, Love G, Studdiford JS (2019). Erythema multiforme: recognition and management. Am Fam Physician.

[4] Sokumbi O, Wetter DA (2012). Clinical features, diagnosis, and treatment of erythema multiforme: a review for the practicing dermatologist. Int J Dermatol.

[5] Aurelian L, Ono F, Burnett J (2003). Herpes simplex virus (HSV)-associated erythema multiforme (HAEM): a viral disease with an autoimmune component. Dermatol Online J.

[6] Siedner-Weintraub Y, Gross I, David A, Reif S, Molho-Pessach V (2017). Paediatric erythema multiforme: epidemiological, clinical and laboratory characteristics. Acta Derm Venereol.

[7] Ginaldi L, Loreto MF, Corsi MP, Modesti M, De Martinis M (2001). Immunosenescence and infectious diseases. Microbes Infect.

[8] Nikolich-Žugich J (2018). The twilight of immunity: emerging concepts in aging of the immune system. Nat Immunol.

[9] Jo N, Hidaka Y, Kikuchi O (2023). Impaired CD4(+) T cell response in older adults is associated with reduced immunogenicity and reactogenicity of mRNA COVID-19 vaccination. Nat Aging.

[10] Moulton VR (2018). Sex hormones in acquired immunity and autoimmune disease. Front Immunol.

[11] Klein SL (2012). Sex influences immune responses to viruses, and efficacy of prophylaxis and treatments for viral diseases. Bioessays.

[12] Kim JY, Lee J, Ko YJ, Shin JY, Jung SY, Choi NK, Park BJ (2013). Multi-indication carbamazepine and the risk of severe cutaneous adverse drug reactions in Korean elderly patients: a Korean health insurance data-based study. PLoS One.

[13] Demouche S, Bettuzzi T, Sbidian E, Laugier Castellan D, Osmont MN, Ingen-Housz-Oro S, Lebrun-Vignes B (2023). Reality of drug-induced erythema multiforme: a French pharmacovigilance study. Therapie.

[14] James F, Goh MS, Vogrin S (2024). The Australasian Registry for Severe Cutaneous Adverse Reactions (AUS-SCAR) - providing a roadmap for closing the diagnostic, patient, and healthcare gaps for a group of rare drug eruptions. World Allergy Organ J.

[15] Yu YM, Shin WG, Lee JY (2015). Patterns of adverse drug reactions in different age groups: analysis of spontaneous reports by community pharmacists. PLoS One.

[16] (2026). Pharmaceuticals and Medical Devices Agency (PMDA). Suspected adverse drug reaction information. https://www.pmda.go.jp/safety/info-services/drugs/adr-info/suspected-adr/0003.html.

[17] (2026). World Health Organization. ATC/DDD toolkit: Anatomical therapeutic chemical (ATC) classification. https://www.who.int/tools/atc-ddd-toolkit/atc-classification.

[18] (2026). International Council for Harmonisation of technical requirements for pharmaceuticals for human use (ICH). Medical Dictionary for Regulatory Activities (MedDRA). https://www.ich.org/page/meddra.

[19] Virtanen P, Gommers R, Oliphant TE (2020). SciPy 1.0: fundamental algorithms for scientific computing in Python. Nat Methods.

[20] Davidson-Pilon C (2019). lifelines: survival analysis in Python. J Open Source Softw.

[21] Phiel KL, Henderson RA, Adelman SJ, Elloso MM (2005). Differential estrogen receptor gene expression in human peripheral blood mononuclear cell populations. Immunol Lett.

[22] Pierdominici M, Maselli A, Colasanti T (2010). Estrogen receptor profiles in human peripheral blood lymphocytes. Immunol Lett.

[23] Matsuda S, Aoki K, Kawamata T (2015). Bias in spontaneous reporting of adverse drug reactions in Japan. PLoS One.

[24] Sugishita K, Maeno M, Tanaka H (2026). Analysis of bevacizumab-related hypertension using the Japanese Adverse Drug Event Report database. BPB Reports.

[25] Sato M, Kageyama K, Ito S (2025). Evaluation of intraocular anti-vascular endothelial growth factor (VEGF) Drug-associated adverse events using spontaneous reporting databases. Cureus.

